# Help-Seeking Intentions and Preferred Sources for Mental Health Problems Among University Students in Saudi Arabia

**DOI:** 10.3390/healthcare14081053

**Published:** 2026-04-15

**Authors:** Yahia Aldhamri, Waleed M. Alshehri, Sara M. Alahmari, Amirah S. Alharbi, Abdullah M. Alanazi, Layla A. Alqahtani, Samya Alshehri, Salman Aloufi, Raeed Alanazi, Ali Kerari

**Affiliations:** 1 Community and Psychiatric Mental Health Nursing Department, College of Nursing, King Saud University, Riyadh 12372, Saudi Arabia; yaldhamri@ksu.edu.sa; 2 Medical Surgical Department, College of Nursing, King Saud University, Riyadh 12372, Saudi Arabia; walshehri1@ksu.edu.sa; 3 College of Applied Medical Sciences, King Saud University, Riyadh 12371, Saudi Arabia; salahmary@ksu.edu.sa; 4 King Khaled University Hospital, King Saud University, Medical City, Riyadh 12372, Saudi Arabia; aalharbi1@ksu.edu.sa; 5 College of Nursing, King Saud University, Riyadh 12372, Saudi Arabia; a.muharib.alanazi@gmail.com (A.M.A.); lalqahtani1@ksu.edu.sa (L.A.A.); asamyh@ksu.edu.sa (S.A.); salofi@ksu.edu.sa (S.A.); 6 Nursing Administration and Education Department, College of Nursing, King Saud University, Riyadh 11421, Saudi Arabia; raalenazi@ksu.edu.sa

**Keywords:** help-seeking, undergraduate students, mental health, stigma, online support, Saudi Arabia

## Abstract

**Highlights:**

**What are the main findings?**

- University students demonstrated a low overall propensity to seek help for mental health problems.
- Students preferred informal and online sources over professional mental health services.

**What are the implications of the main findings?**

- There is a need to enhance mental health literacy, reduce stigma, and strengthen faculty involvement in mental health support in universities.
- Universities should expand online and hybrid mental health services to offer greater access and privacy.

**Abstract:**

**Background:** Mental health problems are highly prevalent among university students in Saudi Arabia; however, help-seeking behaviors remain low despite the availability of mental health services. There is limited evidence regarding students’ intentions to seek help and preferred sources of support, especially formal or informal sources. This study examined help-seeking intentions for mental health problems among university students. **Methods:** This cross-sectional descriptive study was conducted using an online self-administered questionnaire. Participants were 248 undergraduate students from various Riyadh universities. Help-seeking intentions were assessed using the General Help-Seeking Questionnaire. SPSS software was used to perform independent t-tests to assess differences in preferred sources across demographic groups, and Pearson’s correlation analyses were conducted to examine relationships between preferred sources and demographic variables. Multiple linear regression analyses were conducted to examine demographic and academic predictors of intentions to seek help from formal and informal sources. **Results:** Students demonstrated a low overall propensity to seek help. Online sources were the most preferred help-seeking option, followed by mothers, friends, and general physicians, whereas faculty, relatives, and religious persons were the least preferred. Preferred help-seeking sources differed by gender. Seeking help from mental health specialists was positively correlated with age and grade point average. Additionally, the regression analysis for formal help-seeking was significant, explaining 8.4% of the variance, with gender as the only significant predictor. **Conclusions:** These findings suggest the need for targeted interventions to improve students’ help-seeking behaviors. Universities should prioritize mental health literacy initiatives, stigma reduction strategies, and accessible support pathways, particularly by integrating digital and hybrid services and enhancing the role of faculty and institutional support systems in promoting timely and appropriate help-seeking.

## 1. Introduction

Mental health issues among university students are a growing public health priority in Saudi Arabia and worldwide. A report by the Saudi National Mental Health Survey (SNMHS) indicated that among Saudis aged between 15 and 24 years, approximately 40% are at risk of developing mental health issues [1]. Transitioning into adulthood, accompanied by high academic demands and social adjustments, presents university students with substantial challenges that may lead to mental health problems. Emerging evidence from different regions of Saudi Arabia suggests that psychological distress, depression, and anxiety are common among university students. Studies have reported alarming rates of psychological problems among medical students in Saudi Arabia [2,3], with the prevalence of depression ranging from 33.6% to 61.8% [2,4]. Furthermore, an elevated psychological burden has been found among both medical and non-medical undergraduate students [5,6,7]. Despite the availability of effective mental health treatments and support, university students rarely seek help for these issues [8,9,10]. The situation in Saudi Arabia is similar to that in the rest of the world, with low rates of help-seeking for mental health problems [4,8,9,10,11].

Help-seeking refers to individuals engaging in coping behaviors to obtain support from others or through external resources [12]. Help-seeking can be divided into two main types: formal, which includes obtaining help from trained professionals who are recognized and qualified to provide advice, support, or treatment (e.g., psychiatrists and general physicians), and informal, which includes obtaining help from individuals with no professional roles, such as family and friends [12]. The Transactional Model of Stress and Coping provides a useful lens for understanding help-seeking intentions [13]. According to this model, individuals appraise stressful situations and employ coping strategies based on the coping resources available to them. Coping resources refer to personal and interpersonal factors (e.g., social support) that enable individuals to manage challenging situations and shape coping responses, including help-seeking intentions. Consequently, individuals employ coping responses such as seeking help from available sources. Individuals tend to adopt positive coping strategies when coping resources are adequate and choose behavioral disengagement when coping resources are scarce [13,14]. University students may view seeking help from a professional source as a constructive coping response and act based on their coping resources (e.g., availability of services on campus). Students who adopt coping strategies are more likely to develop the intention to seek help from both formal and informal help sources, and those who adopt behavioral disengagement tend to have low intentions to seek help from either source [14].

In addition to informal help sources, university students in Saudi Arabia also have access to various mental health support services, including campus counseling services, university health clinics, and professional mental health practitioners available on- and off-campus. However, despite the existence of multiple formal sources of help, students might prefer to manage their mental health issues privately or confide in family, friends, or religious persons. Although informal help can be valuable, the absence of professional help might worsen problems and affect students’ performance, personal growth, or relationships.

University students’ intentions and behaviors pertaining to seeking help from a specific source depend on various factors. According to Ajzen [15], individuals’ intentions to perform a behavior are influenced by their attitude toward the behavior, subjective norm, and perceived behavioral control. University students’ attitudes toward seeking help from a specific source (e.g., a mental health professional), perceived social expectations from friends, and their perceived ability to obtain support may influence their intentions to seek help. These factors may also shape whether students prefer formal or informal sources of help. Religion, family, and social reputation play important roles in how university students view and respond to mental health issues. This hesitation to seek help stems from several factors, such as the social stigma surrounding mental health issues, self-reliance, and insufficient mental health literacy [4,9,10].

Attitudinal barriers to help-seeking are dominant among Saudi students. Stigma and social embarrassment serve as major barriers and are consistently reported as significant factors preventing Saudi students from accessing formal mental health services [9,10,16,17]. Students might experience embarrassment, fear of other students finding out, and concerns regarding their privacy. Researchers have also reported that Saudi individuals perceive mental health issues as a sign of weakness [9,18,19,20]. Alaqeel et al. [18] reported that university students fear that their visits would be documented in their academic records, which may pose a threat to their academic and career journeys. These findings suggest that stigma and embarrassment arise from deep-rooted cultural beliefs, especially among university students in Saudi Arabia. Mental health issues are often viewed as a source of disgrace rather than a medical condition that deserves formal or professional treatment.

Understanding university students’ help-seeking intentions and their preferred sources remains a critical gap in the Saudi Arabian context. Although several national initiatives have been launched by the Saudi Ministry of Health (MOH) and National Center for Mental Health Promotion (NCMH) to improve mental health awareness, there is a lack of research explaining how university students in Saudi Arabia perceive help-seeking, the sources of help they prefer, their intentions to seek help, and the barriers they face in seeking help for mental health issues. While several studies have examined attitudes toward formal help among Saudi university students, research that systematically distinguishes between formal and informal sources of help within the same sample is limited. Abdulrahman et al. [17] examined planned help-seeking among university students and reported that 53.7% of students planned to seek help for mental health problems; however, they did not distinguish between different sources of help. This limitation highlights the necessity for a comprehensive investigation that concurrently examines intentions across multiple help-seeking sources. This will ensure a clearer understanding of comparative preferences and support patterns among Saudi university students experiencing emotional and psychological distress. Therefore, this study examined help-seeking intentions and preferred sources among university students in Saudi Arabia. The specific aims were:

1. To describe help-seeking intentions for mental health problems among university students.
2. To examine the differences in formal and informal sources of help for mental health problems based on demographic characteristics.

## 2. Materials and Methods

### 2.1. Design

This study employed a cross-sectional descriptive design using an online self-administered questionnaire. Data were collected over a three-month period from October to December 2025. We selected a cross-sectional descriptive design to capture participants’ perceptions and intentions at a single point in time. It is a commonly used design in mental health and behavioral research to examine associations among variables.

### 2.2. Setting and Participants

This study involved undergraduate students enrolled in universities in Riyadh, Saudi Arabia. Riyadh is home to several higher education institutions, including King Saud University, Princess Nourah bint Abdulrahman University, and Imam Mohammad Ibn Saud Islamic University, which are among the largest and oldest universities in the region and offer a wide range of academic programs across health and non-health disciplines.

The target population comprised university students enrolled in bachelor’s degree programs in both health- and non-health-related fields. Participants were recruited using a non-probability convenience sampling technique, which is widely used in survey-based quantitative studies for its feasibility and efficiency in accessing large student populations.

Sample size estimation was conducted using G*Power software (version 3.1) to ensure adequate statistical power. The significance level was set at α = 0.05, with an assumed medium effect size (f^2^ = 0.15). Based on these parameters, the minimum sample size required was 147 participants. Accounting for an anticipated non-response or attrition rate of 20%, the target sample size was increased to 177 participants. Ultimately, 248 completed responses were obtained, exceeding the calculated requirement, thereby enhancing the robustness and generalizability of the findings.

All university students aged 18 years and above who were enrolled in a bachelor’s degree program at the time were eligible to participate. Students who declined to participate or did not complete the questionnaires were excluded from the final analysis.

### 2.3. Measures

#### 2.3.1. Sociodemographic and Academic Characteristics

A structured questionnaire was used to collect sociodemographic and academic information, including age (in completed years), sex, cumulative grade point average (GPA), field of study (health or non-health), academic year (first, second, third, or fourth year or internship/training), history of psychiatric visits, and presence of relatives with mental illness. These variables were included to explore potential differences in help-seeking behaviors across subgroups.

#### 2.3.2. Help-Seeking Intentions and Sources of Help for Mental Health Problems

Help-seeking intentions and sources were assessed using the General Help-Seeking Questionnaire (GHSQ), a validated instrument designed to measure the likelihood of individuals seeking help for mental health concerns [12]. The GHSQ uses a 7-point Likert-type scale ranging from 1 (extremely unlikely) to 7 (extremely likely). It evaluated intentions to seek help from different help sources (i.e., mental health specialists, general physicians, MOH services, and help applications) and informal sources (i.e., mothers, fathers, relatives, friends, intimate partners/spouses, faculty staff, religious people, and online). Higher total scores indicated a greater propensity to seek help. In this study, scores of 5 or higher indicated a high propensity for help-seeking, whereas scores below 5 indicated a low propensity.

GHSQ can be evaluated in various ways based on the research goal. This adaptability highlights the notion that the intentions to seek help are influenced not only by the nature of the issue an individual faces but also by the potential source of assistance [12]. Therefore, scores can initially be assessed separately for each help source. Alternatively, items can be categorized into larger groups of help sources, including formal and informal sources. This adaptable scoring method for GHSQ enables capturing both specific intentions related to sources and the broader trends in formal and informal help-seeking behaviors. In the current study, the overall GHSQ scale demonstrated an acceptable level of internal consistency with an alpha of 0.71. The formal help-seeking subscale achieved a reliability score of 0.72, indicating acceptable consistency. The informal help-seeking subscale had a borderline acceptable reliability score of 0.65.

### 2.4. Data Collection

An English-language web-based survey, comprising the GHSQ along with demographic questions, was conducted following approval from the Institutional Review Board (IRB). The questionnaire was developed using Google Forms, with the first page asking for informed consent and followed by two sections demographics and the GHSQ. The recruitment process involved approaching students through academic groups on social media and the university notification system. Students received an invitation containing a brief explanation of the study’s purpose along with a link to the survey. Participation was voluntary, and respondents could withdraw at any time, without submitting their responses.

### 2.5. Data Analysis

The completed questionnaires were exported for data cleaning, coding, and statistical analysis using the Statistical Package for the Social Sciences (SPSS version 29). Descriptive statistics, including frequencies, percentages, means, and standard deviations, were used to summarize the participants’ characteristics and help-seeking intentions. Independent t-tests were conducted to examine differences in preferred help-seeking sources according to selected demographic and academic variables. The Pearson product–moment correlation coefficient (r) was used to assess relationships between continuous variables. Multiple regression analyses were conducted to identify predictors of help-seeking intentions. The assumptions of inferential statistics (i.e., normality, adequate variance in the independent and dependent variables, absence of influential cases, and linearity) were met. Statistical significance was set at *p* < 0.05.

### 2.6. Ethical Considerations

Ethical approval was obtained from the IRB of King Saud University. This study adhered to the principles of the Declaration of Helsinki. Electronic informed consent was obtained from all participants prior to data collection. Confidentiality and anonymity were ensured by not collecting any personally identifiable information and restricting data access exclusively to the research team. The participants were informed of their right to refuse or withdraw from the study at any time without penalty.

## 3. Results

### 3.1. Participants’ Sociodemographic and Academic Characteristics

A total of 248 university students participated in this study. Descriptive statistics were computed to summarize their sociodemographic and academic characteristics (Table 1). The participants’ mean age was 22.21 years (SD = 3.66). The mean cumulative GPA, on a 5-point scale, was 4.25 (SD = 0.49). The majority of the participants were female (60.9%, n = 151), while 39.1% (n = 97) were male.

In terms of field of study, most students were enrolled in health-related programs (69.8%, n = 173), whereas 30.2% (n = 75) were from non-health fields. Regarding academic level, the largest proportion of participants were in their internship or training year (38.7%, n = 96), followed by the fourth year (23.0%, n = 57), third year (14.5%, n = 36), first year (13.3%, n = 33), and second year (10.5%, n = 26). Regarding mental health-related characteristics, a substantial proportion of participants reported not having visited a mental health specialist previously (75.8%, n = 188). Furthermore, 47.2% of the participants (n = 117) indicated having a friend or relative with a mental illness (Table 1).

### 3.2. Help-Seeking Intentions for Mental Health Problems Across Formal and Informal Sources

On the overall GHSQ, the participants had a mean score of 3.19 (SD = 0.94), indicating a low propensity to seek help when encountering a mental health problem. Regarding subscales, students reported low levels of formal help-seeking intentions, with an average item score of (M = 3.07, SD = 1.55). Conversely, informal help-seeking intentions were slightly higher (M = 3.24, SD = 0.95). Table 2 presents the distribution of help-seeking intentions for mental health problems across formal and informal sources. Overall, a considerable proportion of participants demonstrated reluctance to seek help.

Participants reported being extremely unlikely to seek help from faculty members (60.0%), followed by relatives (55.5%) and religious persons (53.2%). More than one-third of the participants were extremely unlikely to seek help from their fathers (37.6%) and intimate partners or spouses (32.8%). Among formal sources, MOH services and help applications (29.2%) and mental health specialists (21.2%) were extremely unlikely sources of help.

When examining the “very likely” and “extremely likely” sources, online sources (42.4%) emerged as the most preferred help-seeking option for mental health problems. These were followed by mothers (32.4%), general physicians (32.2%), and friends (33.2%). In contrast, very few participants reported being extremely likely to seek help from faculty members (1.2%), relatives (2.8%), or religious persons (3.6%). Among formal sources, 22.4% of the participants preferred seeking help from mental health specialists. Overall, the findings indicated that university students were more inclined to seek help from online platforms, mothers, friends, and general physicians, while showing substantial reluctance to seek help from faculty members, religious persons, or extended family members.

### 3.3. Differences in Preference for Formal Help-Seeking Based on Demographic Characteristics

Independent sample *t*-tests were conducted to examine the differences in the preference for formal help-seeking for mental health problems based on demographic characteristics. Higher total mean scores indicated a greater propensity to seek help for mental health problems. A statistically significant difference was observed between male and female students in seeking help from mental health specialists (t = 3.10, *p* = 0.002): female students reported a higher mean score (M = 4.07, SD = 1.94) than male students (M = 3.24, SD = 2.12). Although female students demonstrated a significantly greater inclination to seek professional mental health support, both groups remained in the low-propensity category. Furthermore, a statistically significant sex-based difference was observed in the preference for the use of MOH services and help applications (t = 2.21, *p* = 0.02); male students reported a lower mean score (M = 2.84, SD = 1.95) than female students (M = 3.41, SD = 2.03) (Table 3).

A statistically significant difference was also observed in the preference for the use of MOH services and help applications based on academic years (t = 2.49, *p* = 0.01). Students in higher academic years reported a higher mean score (M = 3.58, SD = 2.09) than those in lower academic years (M = 2.93, SD = 1.93). However, both groups were classified as having a low propensity to use online help-seeking resources. No significant differences were observed in the preference for general physicians based on sex or academic levels. Additionally, there were no significant differences in the preference for any of the formal help-seeking sources based on the field of study (*p* > 0.05).

### 3.4. Differences in the Preference for Informal Help-Seeking for Mental Health Problems Based on Demographic Characteristics

Male students reported a significantly higher mean score than female students for seeking help from their fathers (t = 3.24, *p* = 0.001; M = 3.53, SD = 2.30 vs. M = 2.64, SD = 1.95). Similarly, a significant sex-based difference was observed for seeking help from relatives (t = 2.27, *p* = 0.007); female students reported a lower mean score (M = 1.99, SD = 1.58) than male students (M = 2.58, SD = 1.77), indicating a very low propensity to seek mental health support from relatives (Table 4).

Statistically significant sex-based differences were observed for seeking help from religious persons (t = 3.43, *p* = 0.001) and online sources (t = 3.30, *p* = 0.001). Female students reported a lower mean score for seeking help from religious persons (M = 2.04, SD = 1.62) than male students (M = 2.82, SD = 1.95); however, both groups indicated a low propensity for help-seeking. Conversely, female students demonstrated significantly greater preference for online help-seeking sources (M = 5.10, SD = 1.88) than did male students (M = 4.25, SD = 2.12).

A statistically significant difference was observed in the preference for seeking help from faculty members based on the study field (t = 2.23, *p* = 0.02). Students enrolled in health-related programs reported a higher mean score (M = 2.05, SD = 1.47) than their counterparts in non-health-related fields (M = 1.62, SD = 1.18). However, both groups were classified as having a low propensity for seeking help from faculty members (Table 5).

### 3.5. Correlations Between Age, GPA, and Help-Seeking Intentions

Pearson’s correlation analyses were conducted to examine the associations between participants’ age, cumulative GPA, and help-seeking intentions for mental health problems. A significant positive correlation was found between age and help-seeking from mental health specialists (*r* = 0.22, *p* < 0.01), indicating that older students had a greater propensity to seek professional support for mental health problems (Table 6).

Similarly, GPA was positively correlated with help-seeking from mental health specialists (*r* = 0.18, *p* < 0.01): students with higher academic performance were more likely to seek help from mental health professionals.

Neither age nor GPA was significantly correlated with other formal sources (general physicians, MOH services, and help applications). Furthermore, none of the informal help-seeking sources (mothers, fathers, relatives, friends, religious persons, faculty members, and online sources) was significantly correlated with either age or GPA (*p* > 0.05).

### 3.6. Predictors of Help-Seeking Intentions

Two multiple linear regression analyses were conducted to examine the demographic and academic predictors of the intentions to seek help from formal and informal sources. The predictor variables included age, study field, sex, academic levels, GPA, previous psychiatric visits, and having relatives with mental illness.

The first regression model examined the predictors of formal help-seeking intentions. This model was statistically significant, F (7, 188) = 2.901, *p* = 0.019. The model accounted for 8.4% of the variance in formal help-seeking intentions. Only gender was a significant predictor of formal help-seeking intentions (β = 0.18, *p* = 0.016). Female students reported higher formal help-seeking intentions compared to male students. However, no statistically significant effects were found for other sociodemographic variables (Table 7).

The second regression model examined the predictors of informal help-seeking intentions. This model was not statistically significant, F (7, 188) = 1.717, *p* = 0.107, and it explained 6% of the variance in informal help-seeking intentions. Academic levels were the only significant predictor of informal help-seeking intentions (β = 0.20, *p* = 0.012). Students in the internship period reported greater intentions to seek help from informal sources compared with those in the first through fourth years of study (Table 8).

## 4. Discussion

This study examined the help-seeking intentions and preferred sources of support for mental health problems among university students in Saudi Arabia. It contributes to the extant literature by distinguishing between formal, informal, and online sources of help, and interpreting these patterns through the Transactional Model of Stress and Coping.

Our findings are consistent with prior research conducted in Saudi Arabia and internationally, which has consistently reported low help-seeking intentions among university students despite the availability of resources [1,8,10,16,18,21,22]. Within the Transactional Model of Stress and Coping, this pattern may reflect constrained cognitive appraisal processes, whereby students perceive their coping resources as limited or inaccessible. Lu et al. [14] demonstrated that behavioral disengagement was negatively associated with both formal and informal help-seeking intentions among first-year Chinese medical students. Cultural norms, stigma, fear of disclosure, and concerns about academic or career consequences may further restrict this appraisal, leading students to delay or avoid seeking help. However, international comparisons have revealed substantial differences in help-seeking intentions across countries, suggesting that structural barriers, such as self-reliance, may operate across diverse cultural settings [11]. These factors may further constrain the appraisal process among university students.

Our study offers a meaningful contribution to the understanding of help-seeking intentions among university students in Saudi Arabia. The current findings align with those of Alaqeel et al. [18] regarding medical students at King Saud bin Abdulaziz University for Health Sciences, where the majority of students expressed reluctance to seek professional help. Nevertheless, this finding contrasts with earlier research conducted in Saudi Arabia, where university students were found to have moderately positive intentions to seek professional help. In addition, Abdulrahman et al. [17] reported that 53.7% of the students at the Imam Mohammed ibn Saud Islamic University in Riyadh had high intentions to seek help. Moreover, a national study across Saudi Arabia revealed a moderate willingness to seek professional help among medical students. The discrepancy between our findings and those of other Saudi studies may be attributed to differences in sample composition, exposure to mental health content within health-related curricula, and place of residence where mental health services are adequately available in Saudi Arabia.

A clear preference for informal and online sources over formal services was observed among university students. In Saudi Arabia, online sources may be appraised as low-risk options that allow individuals to seek support anonymously, thereby preserving privacy and reducing concerns about disclosure. The increasing normalization of digital health services reinforces the acceptability of online help-seeking as a coping strategy, which influences students to adopt a coping strategy involving digital engagement. Ntumi et al. [22] demonstrated that digital engagement weakened the negative effect of stigma on help-seeking behavior among university students. This buffering effect suggests that online platforms expand a student’s perceived coping resource base. Therefore, university students in Saudi Arabia are more likely to rely on sources perceived as emotionally safe, accessible, and socially acceptable [11,19,21,23,24,25,26,27,28]. This is consistent with International evidence reporting positive perceptions of and willingness to utilize online mental health services among university students [29,30,31,32].

Informal sources, particularly parents and friends, were also strongly preferred, reflecting the importance of close interpersonal relationships in shaping coping responses among university students in Saudi Arabia. Abdulrahman et al. [17] found that university students preferred parents (30.2%), friends (22.1%), others (9.2%), and spouses (2.4%) when seeking help for emotional problems. These sources are likely perceived as emotionally supportive and nonjudgmental, which facilitates disclosure [12]. Lu et al. [14] reported that perceived social support indirectly influences help-seeking intentions through active coping strategies. Students who perceive high parental or peer support are more likely to adopt active coping, which in turn leads to higher informal help-seeking intentions [14]. In contrast, formal sources, particularly mental health specialists, may be perceived as less accessible or more stigmatizing as they require explicit acknowledgment of psychological distress. Interestingly, general physicians were more acceptable than mental health specialists, suggesting that students may view non-specialized medical support as a more socially acceptable entry point for care. This highlights the potential role of general physicians as accessible gateways to mental health services within university settings in Saudi Arabia.

Furthermore, our findings indicate that relatives and religious persons were among the least preferred sources of help. In collectivist contexts such as Saudi Arabia, extended family networks may lead students to avoid seeking support from relatives. Similarly, although religious coping may be practiced individually, religious persons may not be perceived as appropriate providers of psychological support [19]. The low preference for religious persons is noteworthy. Students may view religious coping and professional mental health support as separate domains, and they may not consider religious figures as equipped to address psychological problems. This reflects a distinction between religious coping as a personal strategy and religious persons as interpersonal coping resources. However, these findings contrast with those from other cultural contexts, wherein religious and traditional healers are highly preferred [23].

Conversely, our findings suggest that university students may not perceive faculty members as emotionally accessible and acceptable sources of help. This may be explained by university students feeling concerned about confidentiality, fearing academic consequences, or perceiving lack of expertise in addressing mental health issues [18]. This perception may further reduce students’ confidence in faculty as a viable source of support, reinforcing their exclusion from the student’s coping resource network. Such a negative appraisal prevents faculty from inclusion in the student’s coping resource inventory. However, the role of faculty support in student’s mental health in Saudi Arabia is an important area that remains underexplored.

This study revealed significant sex-based differences in help-seeking preferences. Female students showed a higher tendency to seek help from formal sources, including mental health specialists and MOH services and help applications, compared to male students. Female students also demonstrated higher engagement with online help-seeking sources, whereas male students showed a higher tendency to seek help from their fathers. The Transactional Model explains these patterns through gendered differences in coping resource appraisal. Female students may consider a broader range of coping resources as acceptable, including formal services and digital platforms. This broader appraisal reflects high disclosure tendency and greater openness to emotional expression among female students. These findings are consistent with several studies reporting higher help-seeking intentions among female students and stronger self-reliance among male students, which may be explained by gender norms and emotional socialization [23,29,30,33,34]. Stronger endorsement of masculine norms narrows the range of resources male students perceive as acceptable. Saudi students’ adherence to traditional masculine norms may explain why male students are less likely to seek formal help and rely more on informal help sources [35]. Male students who endorse traditional masculine norms may appraise professional services as incompatible with self-reliance, which restricts their coping resource base to only familial and religious sources.

Our results revealed that seeking help from mental health specialists is positively correlated with students’ age and GPA. Older students and those with high academic achievements may exhibit greater awareness, maturity, and autonomy that develops with academic progression. This finding aligns with the Transactional Model’s proposition that coping resources are not static but accumulate and develop over time. As students grow and move forward in their educational journey, they encounter an increasing number of academic challenges. They further develop their comprehension of coping strategies, thereby expanding their array of coping resources. Furthermore, higher GPA may reflect stronger self-regulation. Students with higher academic performance may adopt more proactive coping strategies. These strategies include planning and active problem-solving, which were found to be positively associated with help-seeking intentions [14]. Students who engage in active coping may recognize professional help as a problem-focused response to mental health stressors. This expanded appraisal produces a higher intention to seek help from a mental health specialist as a coping response.

The regression analyses indicated that demographic and academic variables explained only a small proportion of the variance in help-seeking intentions. This finding supports the Transactional Model, indicating that such variables function as distal factors, while cognitive appraisal and perceived coping resources play a more proximal role in determining help-seeking behavior [13,14,18]. Consequently, to better understand help-seeking processes, future research should focus on psychological and attitudinal factors, such as stigma, mental health literacy, and perceived effectiveness of services.

These findings have important practical implications. Universities in Saudi Arabia should prioritize mental health literacy initiatives and stigma reduction strategies to improve students’ perceptions of formal services. Expanding and improving accessibility of digital and hybrid mental health services may align with students’ preferences for online support. Additionally, training faculty members to provide appropriate guidance and ensure confidentiality may help reposition them as supportive resources. At a larger level, policy efforts should focus on developing accessible, culturally sensitive, and confidential mental health services tailored to university students.

This study has several methodological limitations that warrant consideration when interpreting the findings. First, its cross-sectional design limits the ability to establish causal relationships between the variables and restricts temporal inferences regarding help-seeking patterns. Second, reliance on self-report measures may have introduced response bias due to social desirability. Third, the informal help-seeking subscale demonstrated borderline internal consistency (α = 0.65); therefore, findings related to informal sources should be interpreted with caution. Fourth, the use of non-probability convenience sampling limits the generalizability of the findings beyond the sampled universities and regions, and caution should be exercised when applying these results to other student populations in Saudi Arabia or different cultural contexts. Future studies using longitudinal designs, probability sampling, and multi-institutional or national samples are recommended to validate and extend the current findings. Despite these limitations, the present study makes a significant contribution to Saudi mental health literature by providing comprehensive insights into formal, informal, and online help-seeking sources among university students.

## 5. Conclusions

This study’s findings provide significant insights into help-seeking intentions among university students in Saudi Arabia. The overall low help-seeking propensity among university students represents a constrained appraisal process shaped by stigma and cultural norms. Students’ preferred sources of support are those that align with their perceived coping resources. Therefore, informal sources such as parents and friends are appraised as emotionally safe and socially acceptable resources. Formal sources require additional appraisal that includes overcoming stigma and making professional services and faculty support accessible and acceptable. The prominence of online help sources as a preferred source underscores the growing role of digital platforms in students’ help-seeking behaviors. It also highlights the importance of integrating culturally sensitive online mental health services within campuses and the universities’ support systems. Furthermore, the findings emphasize the need to address stigma and gender-related views surrounding mental health issues among male students in Saudi Arabia.

Nevertheless, the findings of this study provide important implications for the mental health of university students. Universities should consider implementing programs that promote mental health services on/off campus and address the stigma of mental health disorders. Additionally, they should consider embedding mental health awareness into curricula and training faculty to recognize and deal with mental health issues. Furthermore, the Ministry of Higher Education should develop comprehensive mental health strategies that integrate accessible online services and the early promotion of counseling during stressful periods such as orientation and exam periods.

The current findings suggest several directions for future research. Future studies should examine psychological and attitudinal barriers, including perceptions of the effectiveness of mental health services. Researchers should also investigate faculty members’ capacity, preparedness, and potential roles in supporting students’ mental health, as these areas warrant closer attention. Furthermore, longitudinal and multisite study designs would be valuable for validating and extending the present findings.

## Figures and Tables

**Table 1:** Sociodemographic and academic characteristics of the participants.

Variable		*n*	Mean (SD)/Percentage
Age		248	22.21 (3.66)
Grade Point Average (out of 5)		248	4.25 (0.49)
Sex	Female	151	60.9%
	Male	97	39.1%
Study Field	Health	173	69.8%
	Non-Health	75	30.2%
Academic Year	First	33	13.3%
	Second	26	10.5%
	Third	36	14.5%
	Fourth	57	23.0%
	Internship/Training	96	38.7%
Previous Psychiatric Visit	Yes	60	24.2%
	No	188	75.8%
Relatives with Mental Illnesses	Yes	117	47.2
	No	131	52.8

SD, standard deviation.

**Table 2:** Help-seeking intentions for mental health problems across formal and informal sources (*N* = 248).

Sources	Extremely Unlikely *n* (%)	Very Unlikely *n* (%)	Unlikely *n* (%)	Neutral *n* (%)	Likely *n* (%)	Very Likely *n* (%)	Extremely Likely *n* (%)
Formal Sources							
Mental Health Specialists	53 (21.1)	34 (13.5)	30 (12.0)	35 (13.9)	42 (16.7)	17 (6.8)	37 (14.7)
General Physicians	35 (14.1)	33 (13.3)	27 (10.9)	31 (12.5)	40 (16.1)	20 (8.1)	62 (25.0)
MOH Services and Help Application	73 (29.1)	43 (17.1)	30 (12.0)	40 (15.9)	23 (9.2)	12 (4.8)	27 (10.8)
Informal Sources							
Intimate Partner/spouse	82 (32.7)	24 (9.6)	18 (7.2)	42 (16.7)	24 (9.6)	24 (9.6)	34 (13.5)
Fathers	93 (37.1)	40 (15.9)	32 (12.7)	22 (8.8)	16 (6.4)	14 (5.6)	31 (12.4)
Mothers	46 (18.3)	29 (11.6)	28 (11.2)	33 (13.1)	32 (12.7)	25 (10.0)	55 (21.9)
Friends	36 (14.3)	33 (13.1)	27 (10.8)	31 (12.4)	40 (15.9)	20 (8.0)	61 (24.3)
Relatives	137 (54.6)	29 (11.6)	28 (11.2)	22 (8.8)	18 (7.2)	8 (3.2)	6 (2.4)
Religious persons	133 (53.0)	27 (10.8)	30 (12.0)	20 (8.0)	16 (6.4)	14 (5.6)	8 (3.2)
Faculty Staff	150 (59.8)	35 (13.9)	27 (10.8)	20 (8)	9 (3.6)	5 (2.0)	2 (0.8)
Online	25 (10.0)	18 (7.2)	26 (10.4)	32 (12.7)	42 (16.7)	31 (12.4)	74 (29.5)

**Table 3:** Differences in the preference for formal sources for help-seeking for mental health problems based on demographic characteristics.

Characteristics	*n*	Mental Health Specialists				General Physicians				MOH Services and Help Applications			
		*M*	*SD*	*t*	*p*	*M*	*SD*	*t*	*p*	*M*	*SD*	*t*	*p*
Sex													
Male	97	3.24	2.12	3.10	0.002	2.19	1.55	1.35	0.17	2.84	1.95	2.21	0.02
Female	153	4.07	1.94			2.50	1.94			3.41	2.03		
Study Field													
Health	174	3.64	1.99	1.21	0.22	2.48	1.77	1.28	0.31	3.17	2.05	0.18	0.85
Non-Health	76	3.99	2.29			2.16	1.86			3.22	1.97		
Academic Year													
Lower Levels	152	3.54	2.12	1.93	0.54	2.32	1.73	0.69	0.48	2.93	1.93	2.49	0.01
Higher Levels	98	4.06	2.01			2.48	1.91			3.58	2.09		

MOH, Ministry of Health.

**Table 4:** Differences in the preference for informal sources for help-seeking for mental health problems based on demographic characteristics.

Characteristics	*n*	Mother				Father				Relatives			
		*M*	*SD*	*t*	*p*	*M*	*SD*	*t*	*p*	*M*	*SD*	*t*	*p*
Sex													
Male	97	4.30	2.18	1.18	0.23	3.53	2.30	3.24	0.001	2.58	1.77	2.27	0.007
Female	153	3.96	2.20			2.64	1.95			1.99	1.58		
Study Field													
Health	174	4.07	2.20	0.25	0.80	3.01	2.17	0.24	0.80	2.25	1.68	0.46	0.64
Non-Health	76	4.14	2.21			2.93	2.07			2.14	1.67		
Academic Year													
Lower Levels	152	4.03	2.17	0.52	0.59	2.84	1.99	1.36	0.17	2.09	1.62	1.49	0.13
Higher Levels	98	3.18	2.24			3.21	2.33			2.42	1.76		

**Table 5:** Differences in the preference for informal sources for help-seeking for mental health problems based on demographic characteristics.

Characteristics	*n*	Faculty Members				Religious Persons				Online Sources			
		*M*	*SD*	*t*	*p*	*M*	*SD*	*t*	*p*	*M*	*SD*	*t*	*p*
Sex													
Male	97	2.04	1.52	1.23	0.26	2.82	1.95	3.43	0.001	4.25	2.12	3.30	0.001
Female	153	1.84	1.32			2.04	1.62			5.10	1.88		
Study Field													
Health	174	2.05	1.47	2.23	0.02	2.33	1.82	0.21	0.80	4.62	2.08	1.75	0.08
Non-Health	76	1.62	1.18			2.38	1.74			5.11	1.84		
Academic Levels													
Lower Levels	152	1.78	1.29	1.87	0.06	2.22	1.68	1.31	0.18	4.86	1.95	0.91	0.36
Higher Levels	98	2.12	1.54			2.53	1.96			4.62	2.12		

**Table 6:** Associations of participants’ age and GPA with formal and informal sources of help-seeking.

	Characteristics			
Formal Sources	Age		GPA	
	*r*	*p*	*r*	*p*
Mental Health Specialists	0.22	<0.01	0.18	<0.01
General Physicians	0.03	0.59	0.04	0.54
MOH Services and Help Applications	0.10	0.11	0.11	0.09
Informal Sources				
Mothers	−0.06	0.37	0.04	0.53
Fathers	−0.02	0.79	0.02	0.71
Relatives	−0.03	0.58	0.01	0.89
Friends	0.03	0.59	0.12	0.07
Religious Persons	−0.09	0.17	0.01	0.93
Faculty Members	0.06	0.36	0.05	0.49
Online Sources	−0.09	0.14	0.04	0.58

GPA, grade point average.

**Table 7:** Multiple linear regression analyses predicting the intentions to seek help from formal sources among university students (*N* = 248).

Predictor	*B^a^*	*SE*	*β^b^*	*t*	*p*-Value	95% CI (*Lower*, *Upper*)
Age	0.150	0.110	0.098	1.366	0.175	−0.067, 0.367
Sex ^a^	1.647	0.677	0.175	2.433	0.015 *	0.311, 2.983
GPA	0.941	0.677	0.099	1.390	0.166	−0.394, 2.276
Academic levels ^b^	0.719	0.742	0.074	0.969	0.334	−0.745, 2.183
Previous psychiatric visits ^c^	−1.286	0.800	−0.116	−1.607	0.110	−2.865, 0.292
Relatives with mental illness ^d^	0.542	0.662	0.058	0.819	0.414	−0.764, 1.849
Study field ^e^	−0.039	0.835	−0.004	−0.047	0.962	−1.687, 1.608
Model summary: R^2^ = 0.084, F (7, 188) = 2.474, *p* < 0.05						

B^a^, unstandardized coefficient; β^b^, standardized coefficient (beta); CI, confidence interval; GPA, Grade Point Average; * *p* < 0.05. ^a^ Sex = male vs. female; ^b^ Academic levels = students in internship period vs. students in first through fourth years; ^c^ Previous psychiatric visits = yes vs. no; ^d^ Relatives with mental illness = yes vs. no; ^e^ Study field = health field vs. non-health field.

**Table 8:** Multiple linear regression analyses predicting the intentions to seek help from informal sources among university students (*N* = 248).

Predictor	*B^a^*	*SE*	*β^b^*	*t*	*p*-Value	95% CI (*Lower*, *Upper*)
Age	0.029	0.188	0.011	0.152	0.879	−0.342, 0.399
Sex ^a^	−1.284	1.158	−0.081	−1.109	0.269	−3.568, 0.999
GPA	1.627	1.157	0.101	1.406	0.161	−0.656, 3.909
Academic levels ^b^	3.219	1.269	0.200	2.538	0.012 *	0.717, 5.722
Previous psychiatric visits ^c^	1.458	1.366	0.078	1.066	0.288	−1.241, 4.156
Relatives with mental illness ^d^	1.050	1.132	0.067	0.928	0.355	−1.183, 3.283
Study field ^e^	0.508	1.428	0.027	0.356	0.723	−2.309, 3.325
Model summary: R^2^ = 0.06, F (7, 188) = 1.717, *p* = 0.107						

B^a^, unstandardized coefficient; β^b^, standardized coefficient (beta); CI, confidence interval; GPA, Grade Point Average. ^a^ Sex = male vs. female; ^b^ Academic levels = students in internship period vs. students in first through fourth years; ^c^ Previous psychiatric visits = yes vs. no; ^d^ Relatives with mental illness = yes vs. no; ^e^ Study field = health field vs. non-health field. * *p* < 0.05.

## Data Availability

The data presented in this study are available on request from the corresponding author due to privacy and ethical restrictions.

## Abbreviations

The following abbreviations are used in this manuscript:

GPA	Grade Point Average
MOH	Ministry of Health
SNMHS	Saudi National Mental Health Survey
NCMH	National Center for Mental Health Promotion
